# Successful fast track protocol implementation for patients undergoing transapical transcatheter aortic valve implantation

**DOI:** 10.1186/s13019-016-0449-4

**Published:** 2016-04-11

**Authors:** Nestoras Papadopoulos, Ali El-Sayed Ahmad, Marlene Thudt, Stephan Fichtlscherer, Patrick Meybohm, Christian Reyher, Anton Moritz, Andreas Zierer

**Affiliations:** Division of Thoracic and Cardiovascular Surgery, Johann-Wolfgang-Goethe University Frankfurt/Main, Theodor Stern Kai 7, 60590 Frankfurt am Main, Germany; Division of Cardiology, Johann-Wolfgang-Goethe University Frankfurt/Main, Frankfurt, Germany; Clinic of Anesthesiology, Intensive Care Medicine and Pain Therapy, Goethe-University Hospital Frankfurt/Main, Frankfurt, Germany

**Keywords:** Heart valve replacement, Transapical, Percutaneous, Heart valve prosthesis (bioprosthetic), Cardiac catheterization/intervention

## Abstract

**Background:**

The aim of the current study is to report our experience with fast-track treatment of patients undergoing transapical transcatheter aortic valve implantation (TA-TAVI) and to determine perioperative predictors for fast-track protocol failure.

**Methods:**

Being one of the pioneering centers to start performing TA-TAVI back in 2005, we routinely included patients undergoing this procedure into our fast-track management program since 2008. Between January 2008 and June 2013, 207 consecutive high-risk patients (mean age 79 ± 7 years, mean Log. EuroSCORE 24 ± 10) who underwent TA-TAVI accordingly to our institutional fast-track approach were prospectively collected and analyzed. Uni- and multivariate analysis were performed to identify independent pre- and perioperative predictors of fast-track protocol failure, defined as inability to discharge the patient from the intensive care unit (ICU) on the day of surgery or as readmission to the ICU 48 h after the initial discharge.

**Results:**

Fast-track management was successful in 83 % of the patients. 30-day mortality was 8 %. Fast-track protocol failure (17 %) was associated with an outcome worsening compared to the remaining patients (mortality: 40 % vs. 2 % and mean hospital stay: 19 ± 12 vs. 10 ± 9 days; *P* = .002). Independent predictors of fast-track protocol failure were age ≥85 years (OR 3.1; CI 95 % 1.89–6.21), ejection fraction (EF) ≤30 % (OR 2.6; CI 95 % 1.99–7.52), moderate to severe preoperative mitral valve regurgitation (OR 2.7; CI 95 % 1.27–6.43) and fluoroscopy time ≥12 min (OR 2.9; CI 95 % 1.28–7.46).

**Conclusions:**

Fast-track patient management following TA-TAVI is safe and reproducible in the majority of patients. Besides patient-related preoperative risk factors (age ≥85 years, EF ≤30 % and moderate to severe preoperative mitral valve regurgitation) a technically challenging intraoperative course as evidenced in a prolonged fluoroscopy time are independent predictors of fast-track protocol failure which is associated with high loss of patient outcome.

## Background

Early pioneer work in 1980s launched the early extubation as a safe technique in a small series of patients undergoing cardiac surgery [1, 2]. Since then, the continuously expanding impact of health economics on the patient management and the frequently limited financial resources have led to an increasing interest in fast-track treatment protocols following cardiac surgery [3].

Transapical transcatheter-based aortic valve implantation (TA-TAVI) has been identified as a safe and efficient alternative to classic surgery, especially in patients carrying an unacceptably high perioperative risk [4, 5]. Due to the minimally invasive nature of this approach that eliminates sternotomy and the need of cardiopulmonary bypass (CPB), TA-TAVI patients may represent a very attractive cohort for fast-track treatment concepts.

Despite the continuous improvement of the surgical and anesthesiological management of patients in cardiac surgery and the insatiable thirst of medical departments for optimized use of intensive care capacities, TA-TAVI patients remain a cohort with high perioperative risk profile and limited biological reserves, who rarely “excuse mistakes” [6, 7]. Thus, the aim of the current study is to report our institutional experience following fast-track treatment of patients undergoing TA-TAVI and to identify predictors for potential fast-track protocol failure.

## Methods

### Patient population

TA-TAVI was introduced as a novel technique in our department in 2005 [8]. Fast-track protocol has been routinely applied for TA-TAVI patients since 2008. Between January 2008 and June 2013, 207 consecutive high-risk patients underwent TA-TAVI followed by the fast-track postoperative treatment approach. The choice of treatment was made at the discretion of the heart team, consisting of 2 cardiac surgeons, 2 interventional cardiologists and lately 2 anaesthesiologists. From 2007 on we assessed all TAVI candidates according to the established recommendations and guidelines in order to find the best possible TAVI approach for each individual patients. Patient data were collected prospectively during treatment using standardized forms to record demographic and clinical characteristics as well as procedural and follow-up data. The local Ethics Committee at the Hospital of the Johann Wolfgang Goethe University, Frankfurt/Main, Germany approved the study protocol and individual patient consent was waived.

### Preoperative anaesthesiologic management

After insertion of a radial artery catheter for invasive monitoring of arterial pressure, anesthesia was introduced using sufentatil (Janssen, Neuss, Germany), disoprivan (Fresenius Kabi, Bad Homburg, Germany) as well as rocuronium (Essex pharma, Munich, Germany), and was maintained with disoprivan (Fresenius Kabi, Bad Homburg, Germany) and remifentanil (Braun, Melsungen, Germany). Oropharyngeal intubation and insertion of a central venous catheter (in the internal jugular vein) was performed afterwards. Mechanical ventilation with biphasic positive airway pressure was established after the intubation adjusted to the gas exchange (Dräger Oxylog 3000, Dräger Medical Germany GmbH, Lübeck, Germany). Further anesthesiologic management included a peripheral venous and urinary catheter. Despite continuous peripheral oxygen saturation control, respiration was monitored at fixed time intervals by blood gas analysis measurements.

### TA-TAVI procedure

Our institutional protocol for TA-TAVI has been previously described in detail [9, 10]. Briefly, all operations were performed in a specially equipped angiography suite that fulfils the standards of a hybrid operating room. Besides standard hemodynamic monitoring, transesophageal echocardiography and CPB were routinely available. A limited left anterolateral incision (5–7 cm), in the fifth intercostal space, was used to access the apex of the heart. A bipolar epicardial pacing wire was placed and tested. Two U stitches with Teflon felt pledgets using 3–0 Prolene polypropylene (Ethicon, Inc., Somerville, NJ) were placed in the apex of the left ventricle. They served as purse string for linear closure of the left ventricle at the end of the procedure. Following balloon valvuloplasty, all valve deployments were performed with standard volumetric inflation of the balloon. Fluoroscopy and transesophageal echocardiography were used to guide the catheter across the native valve and direct deployment of the stent at the level of the annulus. During deployment, the heart was unloaded with rapid ventricular pacing. Valve function was immediately assessed by angiographic and echocardiographic visualization. Intercostal blockade was performed with ropivacaine (Ratiopharm GmbH, Ulm, Germany). The pericardium was partially closed over the apex and a left lateral chest tube inserted. The incision was closed in a standard fashion.

### ICU fast-track protocol

All patients were transferred postoperatively intubated to the intensive care unit (ICU) where ECG, chest radiography and clinical laboratory test as well as blood gas analysis were immediately performed. The ICU is a 36-bed unit run by full-time intensivists and cardiac surgeons with a 2:1 staff to patient ratio. Standard postoperative care consisted of mechanical ventilation, cardioacitve drugs if indicated, the use of warm air heaters to maintain noromothermia and analgesia with a combination of nonsteroidal anti-inflammatory drugs (paracetamol, Ratiopharm GmbH, Ulm, Germany) and intravenous morphine boluses (piritramid, Janssen-Cilag GmbH, Neuss, Germany) as required. Blood gas analyses were repeated half-hourly, the laboratory parameters 4 and 8 h after ICU admission. Hemodynamic monitoring was performed by continuous ECG recording, invasive blood pressure, central venous pressure measurement and monitoring of peripheral and central venous saturation. Mechanical ventilation with biphasic positive airway pressure was turned after the admission to the ICU as soon as possible to continuous positive airway ventilation with low positive end-expiratory pressure. Criteria for weaning from the ventilator included absence of bleeding signs (chest tube drainage <100 ml/h, stable haemoglobin values), stable cardiorespiratory conditions (mean arterial pressure >60 mmHg, central venous saturation >65 and Horowitz index >200), and absence of high inotropic support. If patients fulfilled these criteria, sedation agents were tapered and continuous positive airway ventilation gradually decreased to a minimum level (FiO2: 35 %, PEEP: 5–6 mbar and ASB: 6–7 mbar). Extubation was performed in the presence of appropriate level of consciousness, adequate airway protection reflexes (cough and swallow) and in the absence of respiratory or cardiac distress. Patients were discharged from the ICU to the cardiothoracic ward at least 4 h after extubation but no later than 09:00 p.m. on the day of surgery. During this period, any increasing requirements for cardioactive drugs, or significant decrease in oxygen saturation (<90 % despite oxygen mask), urine output, or level of consciousness, was considered a contraindication for discharge. In the cardiothoracic ward patients monitor surveillance has been performed for two days after the procedure via continuous ECG, peripheral oxygen saturation control and non-invasive blood pressure measurement in a room that fulfils the conditions of an intermediate care unit.

Reasons for readmission included (1) pulmonary (respiratory distress characterized by tachypnea, decrease in arterial saturation <90 %, Horowitz index <200, use of accessory muscles or abdominal paradox, inability to clear secretion); (2) bleeding or pericardial tamponade (new onset bleeding of more than 200 ml/h or more than 800 ml/6 h); (3) severe agitation requiring extended intravenous sedation; (4) upper or lower gastrointestinal bleeding requiring intervention or surgery; (5) any new permanent neurologic deficits (PNDs); (6) hemodynamic instability (any decrease in blood pressure requiring increasing use of cardioactive drugs).

### Data analysis

Data are presented as frequency distributions and percentages. All continuous data are expressed as means ± standard deviation. Categorical data are expressed as counts and proportions. Comparisons were done with 2-tailed *t* test for means of normally distributed continuous variables and the Wilcoxon rank sum test for skewed data. Fisher exact or χ^2^ test was used to analyse differences among categorical data. Univariate and stepwise multivariate logistic regression analysis of perioperative variables for adverse outcome was performed by calculating the odds ratio (OR) with 95 % confidence interval. Fourteen variables were analysed as follows: age older than 80, sex, chronic obstructive lung disease, chronic pulmonary hypertension, arterial hypertension, diabetes mellitus, cerebrovascular disease, peripheral vascular disease, chronic renal insufficiency, smoking history, ejection fraction <30 %, moderate to severe mitral valve regurgitation, previous cardiac surgery and prolonged fluoroscopy time. Statistical analysis of data was conducted with commercially available software (SPSS 20.0 for Windows, SPSS Inc.).

## Results

### Baseline data

Data from all included patients were eligible for further analysis. Mean age was 79 ± 7 years, mean Log. EuroSCORE accounted for 24 ± 10, and 55 % (*n* = 113) of patients were men. Fast-track protocol could be **s**uccessfully applied in 172 patients (83 %) (group S). In the remaining 17 % (*n* = 35) of TA-TAVI patients fast-track management **f**ailed (group F). Demographic data of both groups are summarized in Table 1.

**Table 1:** Baseline demographic and clinical characteristics of group S (successful fast-track protocol) and group F (fast track protocol failure)

	Group S (*n* = 172)		Group F (*n* = 35)		
	No.	%	No.	%	
Variables					*p*
Age (years)	79.2 ± 6.1		86.5 ± 2.6		0.008
Male	88	51	25	71	0.04
STS risk score	8.2 ± 6.8		11.4 ± 8.4		0.18
Log. EuroSCORE	23.2 ± 9.7		25.8 ± 8.3		0.46
Atrial fibrillation	50	29	9	27	0.26
Diabetes	34	20	12	35	0.11
Arterial Hypertension	160	93	29	82	0.21
Pulmonary Hypertension	115	67	26	75	0.25
Previous stroke	26	15	6	17	0.15
Peripheral vascular disease	46	27	14	40	0.08
COPD	53	31	16	47	0.17
Chronic renal failure	91	53	29	82	0.31
Mean ejection fraction (%)	47 ± 15		31 ± 5		0.05
Mean pressure gradient (echo; mmHg)	51 ± 19		44 ± 27		0.14
Aortic valve area (cm)	0.66 ± 0.22		0.72 ± 0.48		0.09
Previous cardiac surgery	50	29	12	35	0.29
Preop. MV regurgitation (moderate to severe)	20	11	12	35	0.05

*COPD* chronic obstructive pulmonary disease, Chronic renal failure = glomerular filtration rate < 60 ml/min/1.73 m^2^; *EuroSCORE* European system for cardiac risk evaluation, *Group F* fast track protocol failure, *Group S* successful fast-track protocol, *MV* mitral valve, *STS risk score* The Society of Thoracic Surgeons’ risk sore

### Postoperative course

**Fig. 1:**
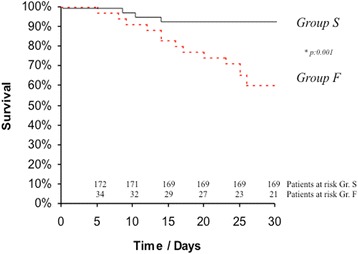
Time to event curves for 30-day survival. Events were calculated with the use of Kaplan Meier methods and were compared using Log rank test. Number of patients at risk is indicated for both groups. Group S denotes patients with successful fast-track protocol after TA-TAVI, whereas Group F represents those patients with fast-track management failure

Overall, 30-day mortality was 8 % (*n* = 17) and the incidence of early PND was 1 % (*n* = 3). Fast-track management failure was associated with a significant dramatic worsening of patient outcome compared to those with successful implementation of fast-track management, as reflected by an increased mortality (group S: 2 % vs. group F: 40 %, *P* = .001) and increased postoperative complication rate such as new renal failure requiring dialysis (group S: 7 % vs. group F: 26 %, *P* = .03). A detailed description of the postoperative course of both groups is presented in Table 2. Figure 1 illustrates the time to event curves for 30-day survival of both groups S and F. The in-hospital stay after failed fast-track protocol was significantly longer (group S: 10 ± 9 days vs. group F: 19 ± 12 days, *P* = .002).

**Table 2:** Detailed postoperative course of group S (successful fast-track protocol) and group F (fast track protocol failure)

	Group S (*n* = 172)		Group F (*n* = 35)		
	No.	%	No.	%	
Variables					*p*
Fluoroscopy time (min)	4.7 ± 2.5		12.9 ± 1.4		0.04
Ventilation time (h)	4.6 ± 1.0		58 ± 15		0.04
Postoperative complications					
- PM-Implantation	9	5	0	0	0.001
- Early-PND	2	1	1	3	0.13
- Major bleeding requiring revision	3	2	10	29	0.02
- Gastrointestinal complications requiring endoscopic intervention	1	1	5	14	0.07
- Acute kidney injury requiring dialysis	12	7	9	26	0.03
Early outcome					
30-day Mortality	3	2	14	40	0.001
Sepsis/MOV	1	1	10	29	
Mesenteric ischemia	1	1	2	6	
Arryythmia	0	0	1	3	
Unknown	1	1	1	3	
Hospital-stay (d)	10 ± 9		19 ± 12		0.002

*Group F* fast track protocol failure, *Group S* successful fast-track protocol, *MOV* Multi organ failure, *PM* Pacemaker, *PND* permanent neurologic deficit

### Causes of fast-track failure

**Fig. 2:**
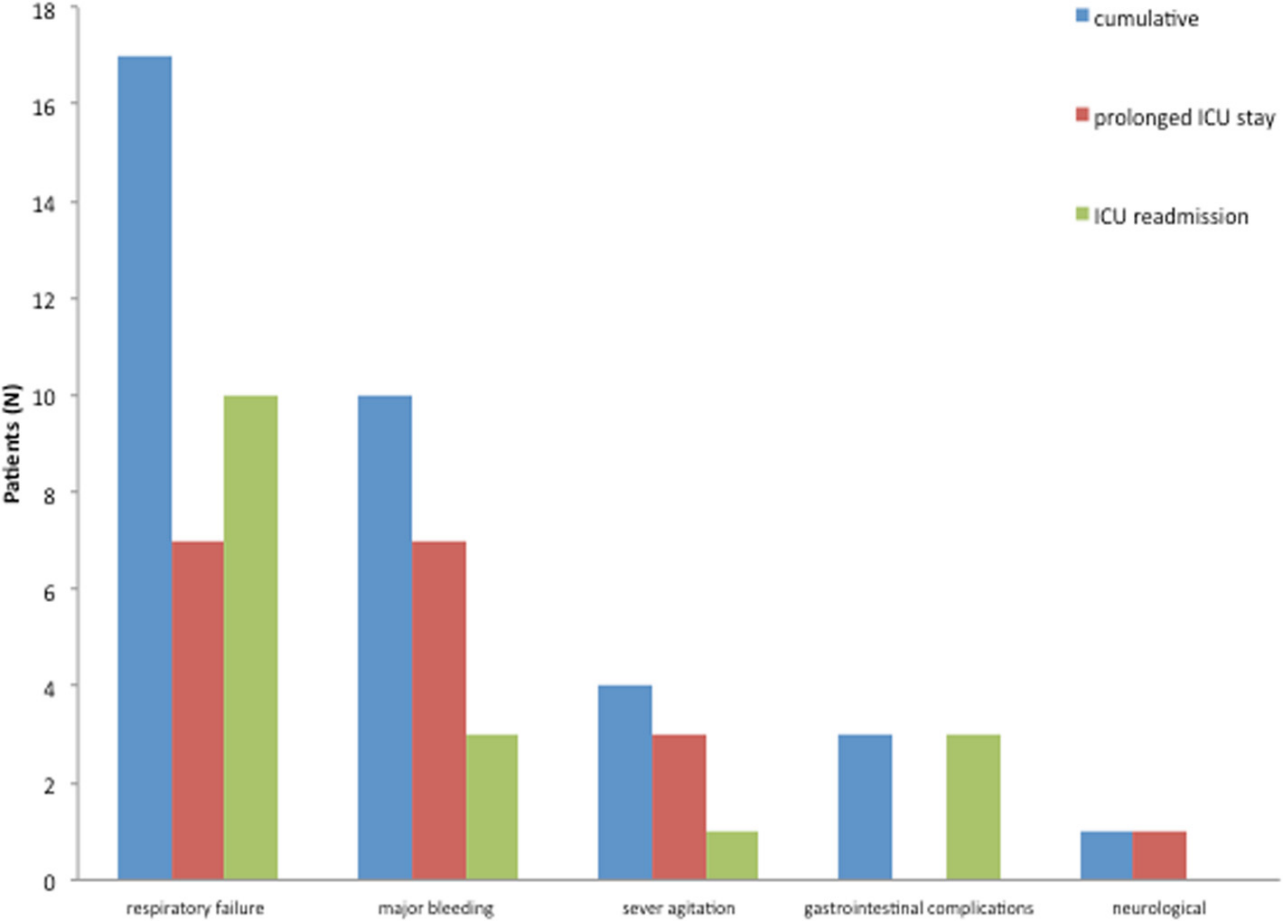
Causes of fast-track failure. ICU = intensive care unit; PND = permanent neurologic deficit

Fast-track protocol failed in 35 patients (group F, 17 %). Twenty-one patients had prolonged ICU-stay and 14 patients required readmission to the ICU within 48 h after initial discharge. Four out of 14 patients, died after readmission to the ICU. The remaining 10 patients could be successfully discharged to the general yard and finally from hospital in the further postoperative course. Reasons for fast-track failure were as follows: respiratory failure (49 %, *n* = 17/35), major bleeding (29 %, *n* = 10/35), severe agitation (11 %, *n* = 4/35), gastrointestinal complications (9 %, *n* = 3/35) and PND (3 %, *n* = 1/35). Figure 2 illustrates the causes of fast-track failure.

### Predictors for fast-track management failure

Independent predictors of fast-track protocol failure were age ≥85 years (OR 3.1; CI 95 % 1.89–6.21, *P* = .004), ejection fraction (EF) ≤30 % (OR 2.6; CI 95 % 1.99–7.52, *P* = .03), moderate to severe preoperative mitral valve regurgitation (OR 2.7; CI 95 % 1.27–6.43, *P* = .008) and fluoroscopy time ≥12 min (OR 2.9; CI 95 % 1.28–7.46, *P* = .04).

## Discussion

Prolonged sedation and ventilatory support have been practiced as an “undisputed standard” since open-heart surgery with use of CPB has been established in the 1950s [11, 12]. Monitoring of potential postoperative complications such as hemorrhage, myocardial ischemia and systemic inflammatory response syndrome following CPB was thought to be adequately performed in sedated patients under stable and easily supervised conditions [12]. Thus, anesthesiologic management with high dose opioid anesthetics made extended ventilatory support of cardiac surgical patients indispensable [13, 14]. However, cost containment and efficient resource use force the pendulum back to the debate of early extubation for patients undergoing cardiac surgery [1–3]. The improvement in anesthesia management coupled with advanced surgical techniques such as minimally invasive cardiac surgery procedures have made early extubation feasible and the concept of balanced anesthesia rather than high-dose narcotics for cardiac procedures has been introduced [4, 15–17]. Nowadays, fast track cardiac anesthesia with early extubation is widely accepted as important step in the recovery process for adult patients [3]. Due to the minimally invasive nature of TA-TAVI that eliminates sternotomy and the need for CPB, TA-TAVI patients represent a particularly attractive cohort for fast-track management concepts [18, 19]. Thus, we sought to investigate the results after routine implementation in our institute with special focus on the risk factors that may predict fast-track protocol failure.

Undoubtedly, our TA-TAVI patients present a cohort with high perioperative risk profile, considering the mean age of 79 ± 7 years and the mean EuroSCORE of 24 ± 10 but despite their limited biological reserves fast-track protocol could be successfully applied in 83 % (Group S) of them. Since, to the best of our knowledge, no available data are published dealing specifically with fast-track management in TA-TAVI patients, only a limited comparison of our results to the literature can be facilitated. Yet, our fast-track protocol success-rate seems to be in line with data published from Haanschoten et al. in 2012, reporting a fast-track protocol success rate by 5367 low risk cardiac surgical patients of 84 % [20]. The ultra short ICU time of Group S could not be translated in a very short hospital stay. Considering the limited biological reserves of this frail patient cohort an average of 10 days hospital stay for completion of physiotherapy treatment, enabling patients of Group F to be discharged either at home or at a rehabilitation center, is not a unique finding in our series. Our data regarding hospital stay are in line with the results reported from Vancouver following TA-TAVI in 178 patients with a mean length of hospital stay of 12 days [21].

The ICU readmission rates 48 h after the initial discharge has been also reported to be 2-5 % for low risk cardiac surgery patients [22–24]. In our series, the readmission rate reached 7 %, which may be seen as a satisfactory result, considering the high-risk profile of our patients. Similar to other reports, the most common reason for ICU-readmission was pulmonary insufficiency [25–27]. Thus, the possibility of providing respiratory support via non-invasive ventilation in a general ward and the active follow-up of TA-TAVI patients after ICU discharge by a dedicated team of respiratory therapists has been identified as an important issue in our clinic. In this context, we believe that avoidance of volume overload in the early perioperative period as well as pain-free management and early mobilisation are of utmost importance for the optimal pulmonary recovery of fast-track TA-TAVI patients.

Another important finding of the current study is that any fast-track management failure is associated with prolonged hospital stay and dramatic worsening of patient outcome. Furthermore, failed fast-track management led to an increased postoperative complication rate such as renal failure requiring dialysis. Although speculative, a possible reason for the high mortality and morbidity in ICU readmission patients may be the early transfer of these patients to the ward. Thus it would be advantageous to identify and focus on subgroups of TA-TAVI patients at risk for fast-track protocol failure. Hence, despite the inherent limitations of a retrospective study, we have identified one procedural (fluoroscopy time ≥12 min) and three preoperative clinical variables to be associated with fast-track protocol failure. Preoperative clinical variables included, age ≥85 years, poor left ventricular ejection fraction (LV-EF ≤30 %) and moderate to severe preoperative mitral valve regurgitation. Recently, D’Onofrio and co-workers reported on the impact of preoperative mitral valve regurgitation on outcomes in 176 consecutive patients undergoing TA-TAVI in a single-centre prospective study setting. They showed that patients with a mitral valve regurgitation ≥2+ had higher surgical risk and a trend towards higher in-hospital mortality [28].

While initial TA-TAVI cases in our series were performed using the first generation of the balloon expandable system significant and relentless technological advances have resulted in new and improved delivery systems and valves. On the other hand at the beginning of the TAVI era, only highest risk patients who were no suitable candidates for open surgery were considered for TAVI. With gaining experience in trancatheter approaches and increased procedural acceptance not only non-surgical-candidates but also high-risk candidates were included in our TAVI-program. Nevertheless a comparison of demographic data and outcomes of the early vs. late TA-TAVI treated patients could not reach a statistical significance in Group F in our series.

## Conclusions

In conclusion, fast-track patient management following TA-TAVI is safe and successful in the majority of patients. Besides patient-related preoperative risk factors (age ≥85 years, EF ≤30 % and severe preoperative mitral valve regurgitation), a technically challenging intraoperative course, as evidenced in prolonged fluoroscopy time, are independent predictors of fast-track management failure, which, in turn, is associated with prolonged hospital stay and dramatic worsening of patient outcome.
